# Assessment of short-term effect of platelet-rich plasma treatment of tendinosis using texture analysis of ultrasound images

**DOI:** 10.2478/raon-2023-0054

**Published:** 2023-11-30

**Authors:** Karlo Pintaric, Vladka Salapura, Ziga Snoj, Andrej Vovk, Mojca Bozic Mijovski, Jernej Vidmar

**Affiliations:** Institute of Radiology, University Medical Center Ljubljana, Ljubljana, Slovenia; Department of Radiology, Faculty of Medicine, University of Ljubljana, Ljubljana, Slovenia; Center of Clinical Physiology, Medical Faculty, University of Ljubljana, Ljubljana, Slovenia; Laboratory for Haemostasis and Atherothrombosis, University Medical Center, Ljubljana, Slovenia; Faculty of Pharmacy, University of Ljubljana, Ljubljana, Slovenia; Institute of Physiology, Faculty of Medicine, University of Ljubljana, Ljubljana, Slovenia

**Keywords:** texture analysis, tendinosis, platelet-rich plasma, ultrasonography

## Abstract

**Background:**

Computer-aided diagnosis (*i.e.*, texture analyses) tools are becoming increasingly beneficial methods to monitor subtle tissue changes. The aim of this pilot study was to investigate short-term effect of platelet rich plasma (PRP) treatment in supraspinatus and common extensor of the forearm tendinosis by using texture analysis of ultrasound (US) images as well as by clinical questionnaires.

**Patients and methods:**

Thirteen patients (7 male and 6 female, age 36–60 years, mean age 51.2 ± 5.2) were followed after US guided PRP treatment for tendinosis of two tendons (9 patients with lateral epicondylitis and 4 with supraspinatus tendinosis). Clinical and US assessment was performed prior to as well as 3 months after PRP treatment with validated clinical questionnaires. Tissue response in tendons was assessed by using gray level run length matrix method (GLRLM) of US images.

**Results:**

All patients improved of tendinosis symptoms after PRP treatment according to clinical questionnaires. Almost all GLRLM features were statistically improved 3 months after PRP treatment. GLRLM-long run high gray level emphasis (LRLGLE) revealed the best moderate positive and statistically significant correlation after PRP (*r* = 0.4373, *p* = 0.0255), followed by GLRLM-low gray level run emphasis (LGLRE) (*r* = 0.3877, *p* = 0.05).

**Conclusions:**

Texture analysis of tendinosis US images was a useful quantitative method for the assessment of tendon remodeling after minimally invasive PRP treatment. GLRLM features have the potential to become useful imaging biomarkers to monitor spatial and time limited tissue response after PRP, however larger studies with similar protocols are needed.

## Introduction

Tendinosis is a broad term encompassing pain and disability at the site of tendons associated with the histopathological findings of failed tendon healing response and no classical signs of inflammation. The most common sites of tendinosis involve the rotator cuff tendons (*i.e.*, supraspinatus tendon), medial and lateral elbow epicondyles (*i.e.*, common extensor tendon), patellar tendon, gluteal tendons and the Achilles tendon).^1^ Several treatment options exist in the process of tendinosis treatment. Conservative options with physiotherapy are widely accepted as the first line therapy, however most provide poor or only empirical evidence.^2^ Surgical management remains the last option due to the morbidity and inconsistent outcomes.

Several minimally invasive treatments with application of different medication have been introduced, providing good patient outcome.^3,4^ Platelet rich plasma (PRP) injection therapy is effective in improving symptoms in supraspinatus tendinopathy and lateral epicondylitis.^5,6^ It is a good treatment option for patients with chronic changes of supraspinatus tendon and lateral epicondylitis who do not meet criteria for surgical management and are not content with the results of conservative treatments.^7^ Platelet-rich plasma is currently one of the most used and investigated therapeutic options in clinical practice to target symptomatic tendinosis, however its effect was mostly monitored only clinically.^8,9^ PRP can be delivered directly into the lesion site and, once activated, the platelet concentrate becomes a gel allowing the secretion of the bioactive molecules in situ and stimulates tendon fibrillar remodulation. PRP is an autologous biotechnology that relies on the local delivery of a wide range of growth factors and cytokines with the aim of enhancing tissue healing.^10^

Gray-scale ultrasound (US) is the most commonly used diagnostic tool to assess tendon pathology, particularly in superficial tendons. It has high level of evidence in shoulder and elbow examination.^11^ It is easily accessible, noninvasive and it has excellent spatial and contrast tissue resolution. It also enables dynamic assessment of tissues as well as can be used repeatedly without potentially harmful effects to the observed tissues. Additionally, it can also be used to guide and assess different treatments applied to tissues.^12^ However, US is a qualitative method and is entirely dependent on the performer with high interobserver variability ranging from poor to good.^13,14^ In order to overcome these limitations and to quantitatively assess tendon structure after specific treatment (*i.e.*, PRP infiltration), several quantitative analyses of tendon texture have already been introduced favoring gray level run length matrix method (GLRLM) due to its highest sensitivity, specificity and accuracy.^15,16^

The aim of this study was to quantify short-term effect of PRP therapy in symptomatic supraspinatus and elbow common extensors tendinosis using texture analysis of ultrasound images.

## Patients and methods

### Study population and study design

Thirteen patients (7 male and 6 female), age 36–60 years (mean age 51.2 ± 5.2) were initially treated with PRP injection therapy for tendinosis at Institute of Radiology, University Clinical Center Ljubljana from March 2019 till March 2020. Four supraspinatus tendons and nine common extensor tendons of the forearm were treated in this study. The inclusion criteria for patients with supraspinatus tendinopathy was confirmed on MRI and persisting pain with disability for at least 3 months in the shoulder after at least one cycle of physiotherapy. The inclusion criteria for patients with lateral epicondylitis was confirmed by common extensor tendinopathy with US and persisting pain with disability for at least 3 months after at least one cycle of physiotherapy. The exclusion criteria were rheumatoid arthritis, known malignancy, other joint injuries found on imaging, bleeding disorders, pregnancy and use of nonsteroidal anti-inflammatory drugs 7 days before PRP treatment as well as previous invasive treatments of the tendons. Patients were evaluated on the day of the PRP injection therapy and at 3 months follow up. The study was approved by the National Medical Ethics Committee of the Republic of Slovenia (approval number 0120 – 115/2018/4). Written informed consent was obtained from the patients or their authorized representatives in accordance with the Declaration of Helsinki.

### Patient evaluation

Standardized questionnaires were used for the assessment of patient clinical outcome. Patients with lateral epicondylitis completed a Patient-rated tennis elbow evaluation (PRTEE) questionnaire, while patients with supraspinatus tendinopathy completed Shoulder pain and disability index questionnaire (SPADI).^17,18^ Both questionnaires were translated into Slovenian language and the scale was adjusted so that 0 points was assessed as the best score (*i.e.*, no pain and no disability) and 100 points as the worst score with severe patient's problems.

### Ultrasound imaging

Real-time US examinations of the affected tendons were performed by one of three radiologists subspecialized in musculoskeletal imaging with at least 5 years of experience. US examinations were performed using a 13–15 MHz electronic linear-array transducer on a ProSound F75 scanner (Hitachi Aloka Medical, Ltd. Tokyo, Japan). The imaging resolution was approximately 1 mm in the longitudinal plane, scan width 38 mm and depth of field approximately 50 mm. Six consecutive US images of the tendons were acquired comprising the most affected regions of tendinosis. Probe was placed longitudinally along the course of the tendon fibers with minimal probe movement to acquire the images. At the follow up, the second set of US images were acquired in the same manner. Patient's bony and soft tissue landmarks were used to obtain comparable imaging location, angle and plane.

### PRP injection therapy

Platelet-rich plasma preparation was obtained from patient autologous blood. Approximately 30 mL of blood was drawn from patient's non-treated arm and mixed with 1 ml of anticoagulant solution (citrat dextrose). Centrifugation was performed using Harvest SmartPrep 2 (Terumo BCT, USA), which yielded approximately 3 mL of PRP. PRP injection therapy was performed under US guidance into the affected tendon. Patients were in supine position, surgical disinfection was performed and sterile probe sleeve was used. 3 mL of local anesthetic (Xylocaine^TM^) was infiltrated in subacromialsubdeltoid bursae in patients treating supraspinatus tendinosis and in subcutaneous tissue around common extensor tendons in patients treating lateral epicondylitis.^19,20^ Approximately 2 ml of PRP was injected in the most heterogeneous area of the tendon on B mode. Small amount of PRP, which was not applied into the treated tendons, was analyzed in the Laboratory for Haemostasis and Atherothrombosis (University Medical Centre, Ljubljana, Slovenia) for PRP compounds (*i.e.*, platelet and leukocyte concentration).

### Image and statistical analysis

**FIGURE 1.**
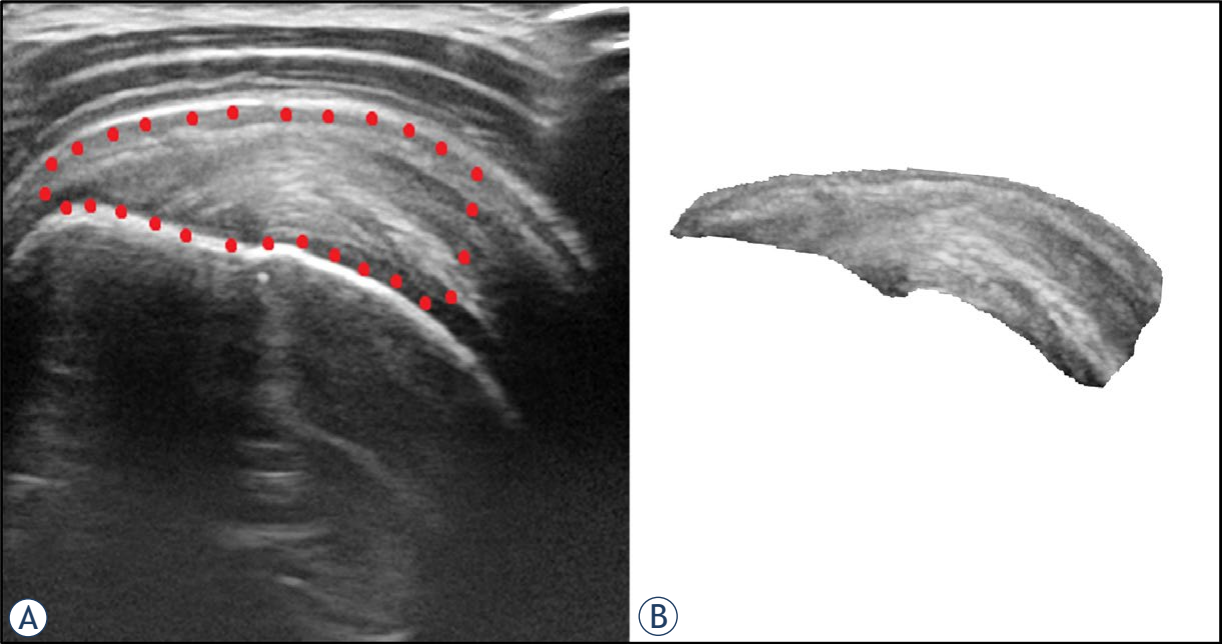
US image of the representative supraspinatus tendinosis in longitudinal plane **(A)**. Region of interest was selected from the footprint of the tendon to the myotendinous junction of supraspinatus muscle (encircled with red dots) and cropped for the purpose of texture analysis **(B)**.

Open source program for image processing, ImageJ (NIH programs, USA) was used. Region of interest (ROI) was selected in each 8-bit DICOM gray scale image from the set of 6 consecutive slices of each affected tendon as shown in Figure 1. Extracted ROI were then analyzed within statistical program R v4.1.2 (R Core Team, Austria). Texture features extraction based on gray level run length matrix (GLRLM) were done with radiomics Image Processing Toolbox inside the R (Radiomics v 0.1.3) Statistical analysis involved mixed effect statistical modeling with the help of R package NLME *i.e.* (non-linear model effect, v 3.1–155). The assumptions about collinearity and homoscedasticity were checked with residual and Q-Q plots. To achieve the normal distribution of data and to avoid outliers we selected maximum run length of 5 inside the GLRLM feature algorithm calculation of the study. Eight GLRLM features were extracted: grey level non-uniformity (GLN), long run emphasis (LRE), high gray level run emphasis (HGLRE), long run high gray level emphasis (LRHGLE), long run low gray level emphasis (LRLGLE), low gray level run emphasis (LGLRE), run length nonuniformity (RLN) and run percentage (RP). Each of the features was tested for ability of prediction of tendon remodeling and clinical outcomes as assessed by questionnaires and *p*-value below 0.05 was considered as statistically significant.

## Results

All patients stated at least some improvement of symptoms after PRP injection therapy. Laboratory analysis of PRP showed, that applied PRP consisted of average platelet concentration of 1954 × 10^3^/μL (range 1524 × 10^3^/μL – 2163 × 10^3^/μL) and average leukocyte concentration of 24 × 10^3^/μL (range 15,6 × 10^3^/μL – 31,5 × 10^3^/μL). Median score at PRTEE questionnaire for patients with epicondylitis before treatment was 65 (range 36 – 79), after treatment 26 (range 3 – 57) with a median improvement in score of 28. Median score at SPADI questionnaire for patients with shoulder problems before treatment was 47 (range 16 – 75), after treatment 12 (range 0–70) with a median improvement in score of 18.

**FIGURE 2.**
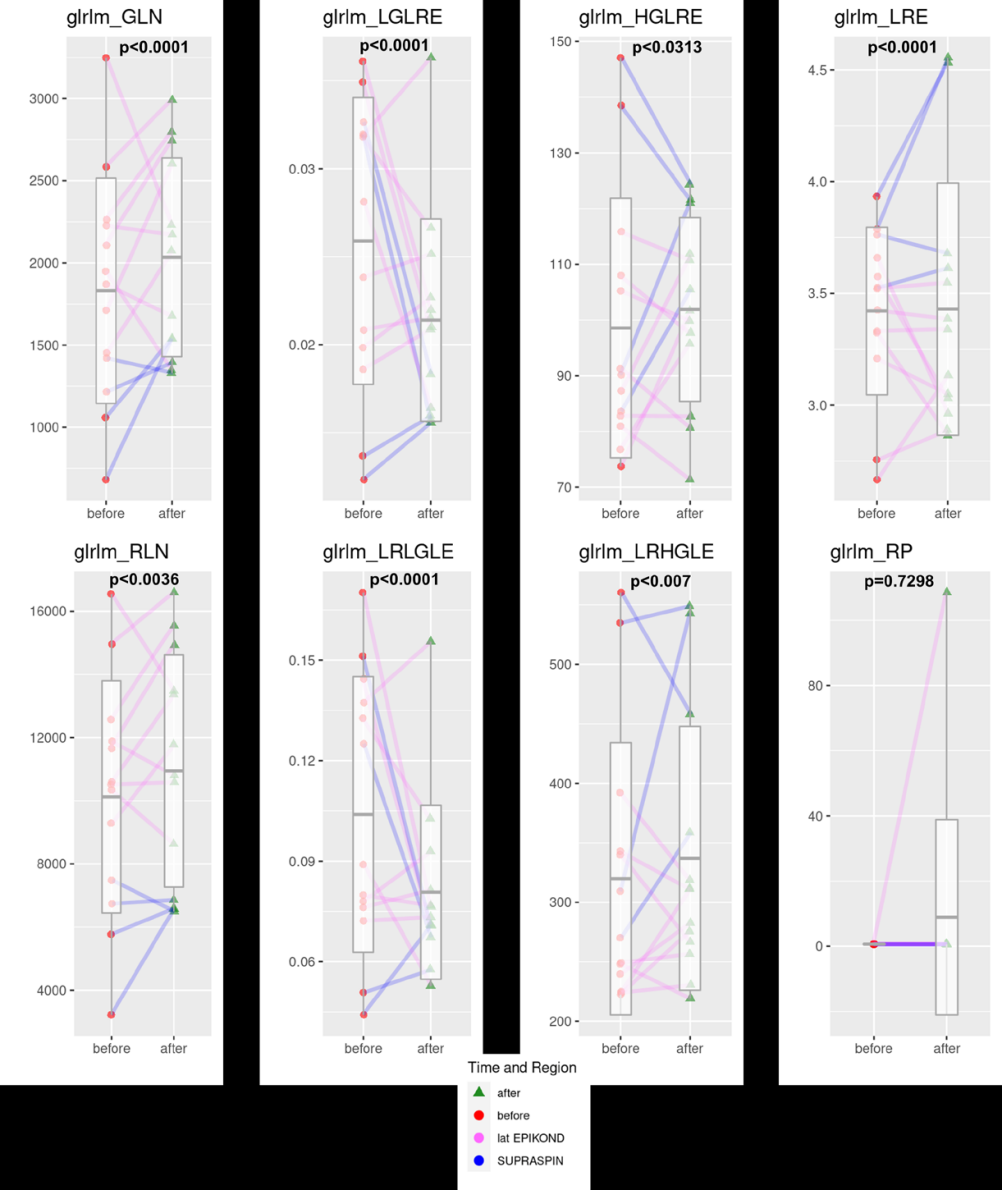
Box plots of the amplitude using gray level run length matrix method (GLRLM) features before and after platelet rich plasma (PRP) treatment (supraspinatus tendons encoded with blue and common extensor tendons encoded with pink).

Figure 2 shows detailed statistical analysis of eight GLRLM features in treated tendons (*i.e.*, common extensor tendons and supraspinatus tendon) three months after PRP treatment. As can be seen from the Figure 2 values of almost all used GLRLM features were statistically significant after PRP treatment except for GLRLM-RP. Additionally, analysis of GLRLM features also showed that changes of their values occurred in similar trends in both studied tendon groups, *i.e.*, supraspinatus (colored in blue) and common extensor tendon group (colored in green).

**FIGURE 3.**
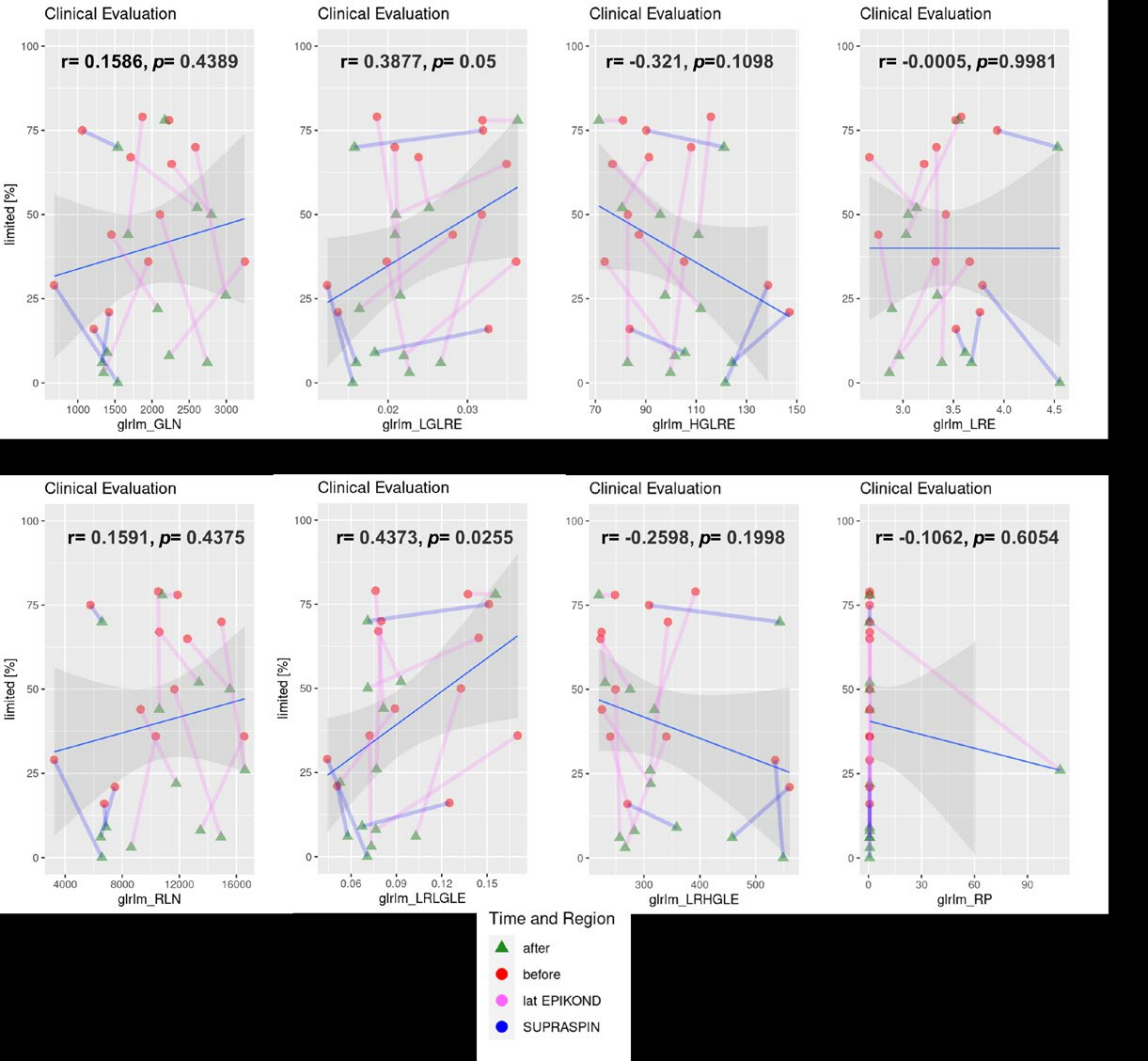
Clinical evaluation dependency of average texture feature per subject divided by tissue region and time. Tissues are coded with blue and pink lines, for supraspinatus and common extensor, while the time of measurement is indicated by a red circle for the first measurement before treatment and a green triangle for the measurement after treatment.

Correlations according to the mixed effect model between clinical evaluation and changes in GLRLM features are shown in Figure 3. In the analysis it is seen that between among all used features GLRLM-LRLGLE has moderate positive and statistically significant correlation with clinical evaluation after PRP (*r* = 0.4373, *p* = 0.0255). Weak positive correlation is also seen for GLRLM-LGLRE (*r* = 0.3877, *p* = 0.0500) as well as weak negative correlation is seen for GLRLM-HGLRE, however, the later without statistical significance (*r* = −0.3210, *p* = 0.1098). Other used features showed weak correlations (*i.e.* positive as well as negative) but, also, without any statistical significance.

## Discussion

This study aimed to perform texture analysis of symptomatic tendinosis US images of supraspinatus and elbow common extensors tendons after PRP treatment. Several GLRLM features were analyzed and found that almost all used features were significantly improved after PRP treatment.

Computer-aided diagnosis tools (*i.e.*, texture analyses) are becoming increasingly beneficial methods to monitor subtle tissue changes, *i.e.*, small tendon tears or tissue repair. We used tools for segmentation, texture analysis and area computation, which allowed us to increase the accuracy and more quantitative analysis of the changes in the tendons, otherwise hardly recognized with our eyes. Texture analysis enabled to quantitatively monitor the PRP effect in tendons. Almost all the applied features enabled to quantify intuitive qualities due to tissue changes in the observed tendons already shortly after PRP as a function of the spatial variation in pixel intensities of US images. Eight GLRLM features were used since it was already shown that this set of GLRLM features provides optimal sensitivity, specificity, and accuracy to characterize tissue properties.^16^

In our study most of the values of GLRLM features were significantly altered already in short-time interval (*i.e.*, 3 months post PRP), as well as almost all treated patient reported good symptomatic improvements of tendinosis. Although the process of tissue repair can take up to 12 months, we have already quantitatively observed significant tissue changes shortly after PRP. However, the most significant correlation was obtained only in features, which are linked to longitudinal oriented echotexture of the treated tendons. This seem reasonably since longitudinal reorientation is most comparable to architecture of normal tendon. Specifically, GLRLM-LGLRE showed moderate positive correlation and GLRLM-HGLRE feature showed weak positive correlation with clinical questionnaires. Both of these features describe changes of low gray level emphasis, which in our case turned out to be the most important parameter of texture analysis to monitor progress of tissue response. In comparison, GLRLM-LRLGLE was favored since it depicted tissue changes (*i.e.*, reduction of hypoechoic areas) in a longitudinal direction. Our results seem to be in accordance with previous studies, which reported similar reduction of hypoechoic areas in tendinosis after PRP application, however these observations were semiquantitative and not linked to texture orientation.^19,20^ In a normal tendon the fibers are usually arranged longitudinally in a fibrillar pattern, forming a relatively homogeneous tissue texture. In the tendinosis, the fibers lose their longitudinal fibrillar pattern and tissue texture turns out to be more heterogeneous and deranged. In the normal healing process of the tendons the fibers remodel their orientation again more longitudinally.^21^ In our study similar alterations of the fibrillar pattern were observed after PRP treatment.

So far, several studies have been performed to follow-up the effect of various treatment options of tendinopathies. However, mostly these were semi-quantitative evaluations based on standard US criteria for tendinopathy, such as focal or diffuse loss of uniform tendon echotexture, thickening of the tendon, loss of fibrillar structure and neovascularity.^22^ In our study GLRLM avoids semi-quantitative assessment of tendinopathy based on “classic” US features and enabled to quantify properties of the tendon by their texture content. This approach enabled us to analyze tissue imaging and clinical outcome after PRP more objectively.

Despite still uncertain steps of PRP effects, in the short-term the factors released by the platelets most likely lower tissue inflammation and modulate the pain receptors sensibility.^23^ Afterwards, PRP favors the cell proliferation with collagen and matrix deposition, as well as tissue remodeling. The final outcome is tissue scar formation, which can help to restore tendon function.

In the study, two groups of tendons were treated (*i.e.*, symptomatic supraspinatus and common extensor tendinosis) without any major differences between the groups comparing the initial or post-treatment texture analyses. This could be attributed to a small number of subjects and consequently making difficult to observe any major consideration about the differences between tendon groups. On the other hand, we hypothesized that similar, spatial and time related tissue changes occurred in the both tendon groups before and after PRP, which are not significantly discernable by texture analysis. Tendons with potential to assess its remodeling after PRP treatment with GLRLM are preferably superficially lying tendons like rotator cuff tendons, common extensor and flexor tendons of the forearm, patellar ligament, where high-resolution US images with optimal signal-to-noise ratio (SNR) could be obtained in a repetitive manner. Deeply lying tendons with complex anatomical structures *e.g.*, proximal hamstrings tendons, are probably less suitable for texture analysis due to the lack of optimal SNR which is a basic prerequisite for texture analysis.

All treated patients improved of tendinosis symptoms in rather short time prior to complete tendon remodeling. This effect could be associated also with high leukocyte concentration of the obtained PRP. We assumed that the leukocyte-rich content of PRP influenced the concentration of various cytokines and modulate local immune and inflammatory response similar as reported in previous studies.^24^

The study had several limitations. Firstly, the studied sample in this pilot study is rather small since some of the treated patients did not return to the follow up. Secondly, the observation period was relatively short. Although we observed clinical improvements after three months, remodulation of the treated tendons was probably not yet completed in this short period of time by means of texture analysis. Therefore, the next step would be to perform a similar study in a larger group with intermediate as well as longer observation time period using a comparable concept of texture analysis and clinical questionnaires. In the study the intraobserver variability was also addressed. It was lowered as much as possible by taking multiple consecutive slices of each tendon and placing the US probe in the similar positions regarding bony and soft tissue landmarks at the initial and posttreatment imaging. Finally, we studied only the effect of leukocyte-rich PRP compound and did not make any comparison between different PRP compound, *i.e.*, leukocyte-poor PRP versus leukocytes-rich PRP.

In conclusion, computer aided texture analysis of US tendinosis enabled to quantify tendon remodeling in short time period after PRP treatment, with the most pronounced effect in features, sensitive to changes in longitudinal tissue orientation. Features of GLRLM analysis have the potential to become useful imaging biomarkers to monitor spatial and time limited tissue response, however larger studies with similar protocols are needed.

## References

[1] Millar NL, Silbernagel KG, Thorborg K, Kirwan PD, Galatz LM, Abrams GD (2021). Tendinopathy. Nat Rev Dis Primers.

[2] Kuhn JE (2009). Exercise in the treatment of rotator cuff impingement: a systematic review and a synthesized evidence-based rehabilitation protocol. J Shoulder Elb Surg.

[3] Sconfienza LM, Adriaensen M, Albano D, Allen G, Aparisi Gomez MP, Bazzocchi A (2020). Clinical indications for image-guided interventional procedures in the musculoskeletal system: a Delphi-based consensus paper from the European Society of Musculoskeletal Radiology (ESSR)-part I, shoulder. Eur Radiol.

[4] Sconfienza LM, Adriaensen M, Albano D, Aparisi Gomez MP, Bazzocchi A, Beggs I (2020). Clinical indications for image-guided interventional procedures in the musculoskeletal system: a Delphi-based consensus paper from the European Society of Musculoskeletal Radiology (ESSR)-Part II, elbow and wrist. Eur Radiol.

[5] Chianca V, Albano D, Messina C, Midiri F, Mauri G, Aliprandi A (2018). Rotator cuff calcific tendinopathy: from diagnosis to treatment. Acta Biomed.

[6] Ma KL, Wang HQ (2020). Management of lateral epicondylitis: a narrative literature review. Pain Res Manag.

[7] Giovannetti de Sanctis E, Franceschetti E, De Dona F, Palumbo A, Paciotti M, Franceschi F (2020). The efficacy of injections for partial rotator cuff tears: a systematic review. J Clin Med.

[8] Kearney RS, Ji C, Warwick J, Parsons N, Brown J, Harrison P (2021). Effect of platelet-rich plasma injection vs sham injection on tendon dysfunction in patients with chronic midportion achilles tendinopathy: A randomized clinical trial. JAMA.

[9] Rha DW, Park GY, Kim YK, Kim MT, Lee SC (2013). Comparison of the therapeutic effects of ultrasound-guided platelet-rich plasma injection and dry needling in rotator cuff disease: a randomized controlled trial. Clin Rehabil.

[10] Everts P, Onishi K, Jayaram P, Lana JF, Mautner K (2020). Platelet-rich plasma: new performance understandings and therapeutic considerations in 2020. Int J Mol Sci.

[11] Sconfienza LM, Albano D, Allen G, Bazzocchi A, Bignotti B, Chianca V (2018). Clinical indications for musculoskeletal ultrasound updated in 2017 by European Society of Musculoskeletal Radiology (ESSR) consensus. Eur Radiol.

[12] Tumpaj T, Potocnik Tumpaj V, Albano D, Snoj Z (2022). Ultrasound-guided carpal tunnel injections. Radiol Oncol.

[13] Cavaggion C, Navarro-Ledesma S, Luque-Suarez A, Juul-Kristensen B, Voogt L, Struyf F (2023). Subacromial space measured by ultrasound imaging in asymptomatic subjects and patients with subacromial shoulder pain: an inter-rater reliability study. Physiother Theory Pract.

[14] Ingwersen KG, Hjarbaek J, Eshoej H, Larsen CM, Vobbe J, Juul-Kristensen B (2016). Ultrasound assessment for grading structural tendon changes in supraspinatus tendinopathy: an inter-rater reliability study. BMJ Open.

[15] Paris MT, Mourtzakis M (2021). Muscle composition analysis of ultrasound images: a narrative review of texture analysis. Ultrasound Med Biol.

[16] Park BE, Jang WS, Yoo SK (2016). Texture analysis of supraspinatus ultrasound image for computer aided diagnostic system. Healthc Inform Res.

[17] Breckenridge JD, McAuley JH (2011). Shoulder pain and disability index (SPADI). J Physiother.

[18] Rompe JD, Overend TJ, MacDermid JC (2007). Validation of the patient-rated tennis elbow evaluation questionnaire. J Hand Ther.

[19] Filardo G, Kon E, Di Matteo B, Pelotti P, Di Martino A, Marcacci M (2013). Platelet-rich plasma for the treatment of patellar tendinopathy: clinical and imaging findings at medium-term follow-up. Int Orthop.

[20] Finnoff JT, Fowler SP, Lai JK, Santrach PJ, Willis EA, Sayeed YA (2011). Treatment of chronic tendinopathy with ultrasound-guided needle tenotomy and platelet-rich plasma injection. PM R.

[21] Cook JL, Purdam CR (2009). Is tendon pathology a continuum? A pathology model to explain the clinical presentation of load-induced tendinopathy. Br J Sports Med.

[22] Docking SI, Ooi CC, Connell D (2015). Tendinopathy: is imaging telling us the entire story?. J Orthop Sports Phys Ther.

[23] Abate M, Verna S, Di Gregorio P, Salini V, Schiavone C (2014). Sonographic findings during and after platelet rich plasma injections in tendons. Muscles Ligaments Tendons J.

[24] Kobayashi Y, Saita Y, Nishio H, Ikeda H, Takazawa Y, Nagao M (2016). Leukocyte concentration and composition in platelet-rich plasma (PRP) influences the growth factor and protease concentrations. J Orthop Sci.

